# Anesthesia for penetrating injury of the oropharynx in a child: a case report

**DOI:** 10.1186/s42077-023-00325-7

**Published:** 2023-05-09

**Authors:** Pallav Bhandari, Ruchi Kapoor, Sujata Chaudhary, R Lakshmi

**Affiliations:** 1 Department of Anesthesiology and Critical Care, University College of Medical Sciences and Guru Teg Bahadur Hospital, Shahdara, Delhi 95, India; 2 Gurgaon, India

**Keywords:** Toothbrush, Gingivobuccal sulcus, Fibreoptic, Videolaryngoscope

## Abstract

**Background:**

Penetrating injury of the oropharynx occurs frequently in children, however, anesthetic management is seldom described in such cases.

**Case presentation:**

A 2-year old child came to the emergency room with a toothbrush impacted in the gingivobuccal sulcus making airway management difficult. We used a simple yet unique approach to secure the airway safely given the lack of pediatric size fibreoptic and videolaryngoscopes in our emergency operation theatre. The patient was kept in Pediatric ICU and watched for any complications and discharged on the 4th postoperative day.

**Conclusions:**

Thus, ingenious non-invasive techniques to secure the airway can prevent the patient from undergoing surgical tracheostomy.

## Background

Penetrating injuries of the oropharynx occur frequently in children less than 4 years of age, with a wide array of objects such as pens, pencils, cylindrical toys, sticks and straws, and rarely with a toothbrush too. The foreign object can lodge in the posterior pharyngeal wall, soft and hard palate, tonsillar region and uvula (Kupietzky 2000; Younessi and Alcaino 2007). They are usually removed by the parents, few make it to the emergency room thus exact incidence is difficult to predict however it is two to three times more common in males than females. If in proximity to internal carotid artery (ICA), various life-threatening complications can occur like ICA thrombosis and mediastinitis (Kosaki et al. 1992; Incollingo and Shevchenko 2007). If such a patient presents to the anesthesiologist, a thorough evaluation of the airway is essential considering poor patient cooperation, risk of bleeding, aspiration, and anticipated difficult airway alongside the challenges of pediatric anesthesia. The injury may seem innocuous but may be associated with pressure symptoms due to direct compression or thrombosis of internal carotid artery which is in proximity. We report one such rare case which presented in emergency and was successfully managed while avoiding invasive airway management.

## Case presentation

**Fig. 1:**
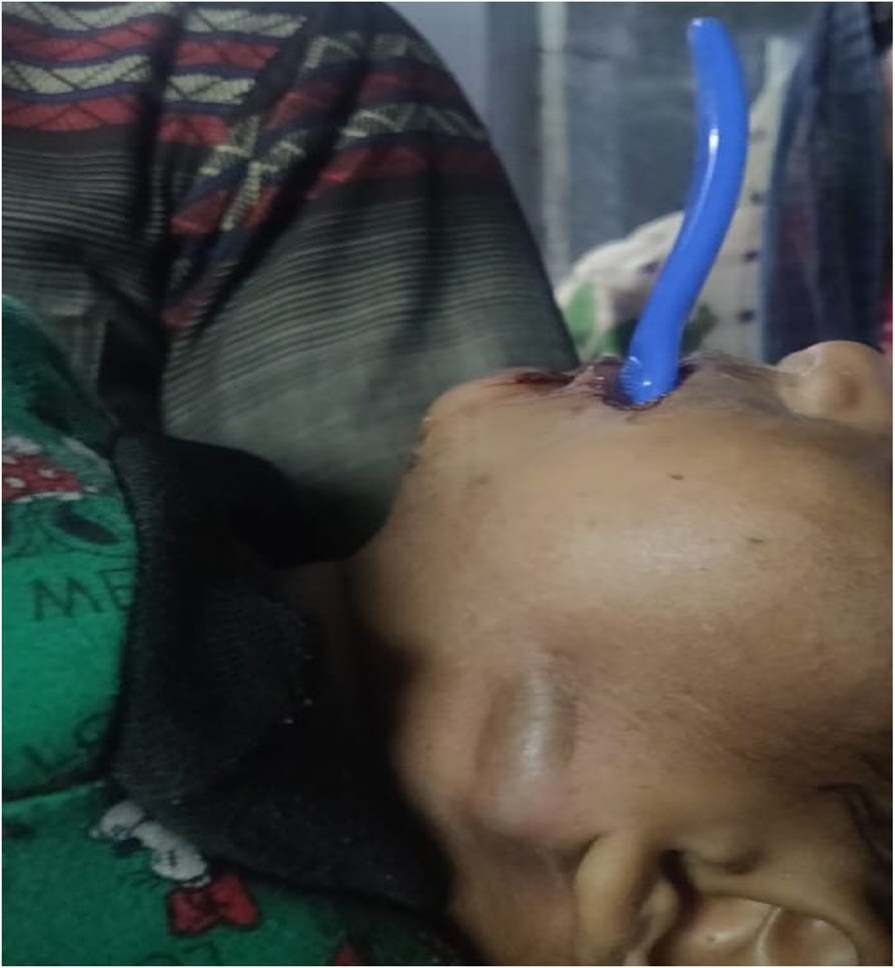
Toothbrush embedded in gingivobuccal sulcus (side view)

A 2-year old normal healthy male child, weighing 10.2 kg presented to the emergency room with a history of impacted toothbrush, seven hours after he fell face-first onto the ground while brushing his teeth (Fig. 1).

There was no ENT bleed and no signs of respiratory distress, therefore surgeons attempted to remove the toothbrush in the emergency room but they were unsuccessful; so, it was decided to remove the impacted toothbrush via intraoral approach under general anesthesia.

A thorough clinical assessment of the child was done while the operation room was being prepped up. Routine investigations including rapid antigen test (RAT) for COVID-19 were asked for. Postoperative ventilation consent and high-risk consent and tracheostomy consent were taken.

When seen by the anesthesiologist in the preoperative room, the child was conscious but exhausted. The patient had a blood pressure of 90/60 mmHg and pulse rate of 130/min, respiratory rate of 28–32 cycles/min and Spo2 of 99% on room air. General physical examination was unremarkable. Oral examination revealed a toothbrush of 15 cm in length impacted in left gingivobuccal sulcus 2 cm above and behind the first premolar with 10 cm distal portion of brush protruding out of the oral cavity. Excessive salivation was present. Bulge due to the bristle side of the brush could be seen in the retromandibular area on the outside.

**Fig. 2:**
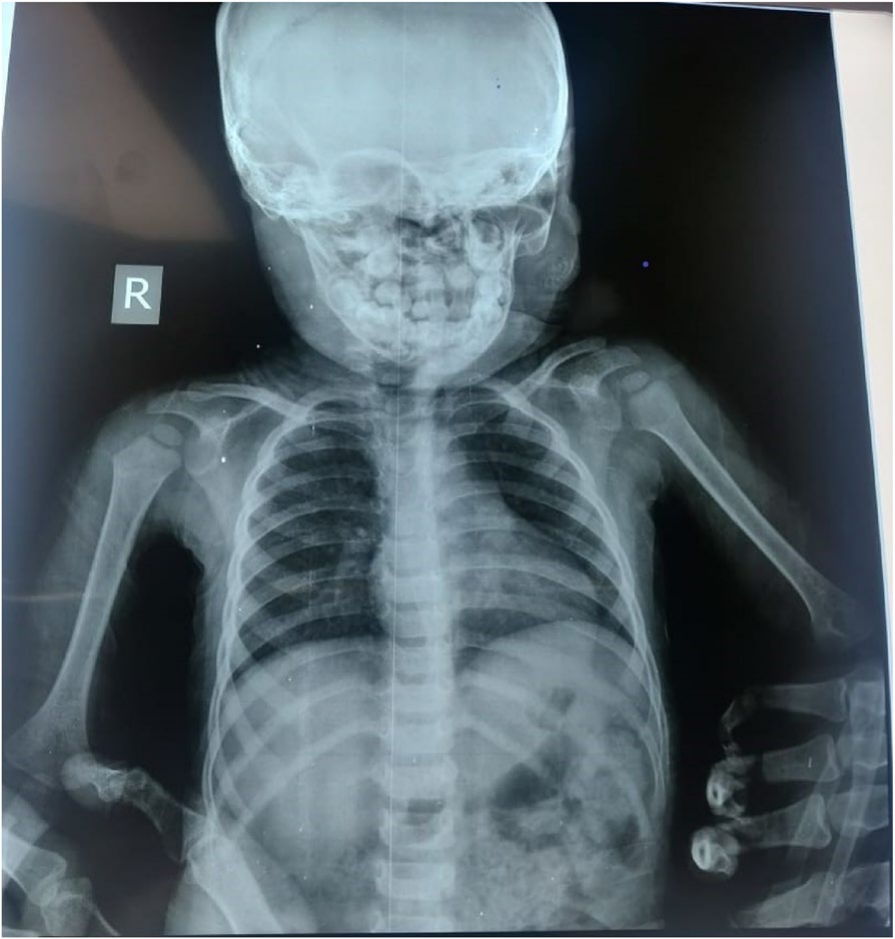
X-ray AP view showing position of toothbrush

Routine investigations were within normal limits RAT was negative for COVID-19. X-ray skull AP view revealed a radiolucent body on the left side lateral to the mandible (Fig. 2).

**Fig. 3:**
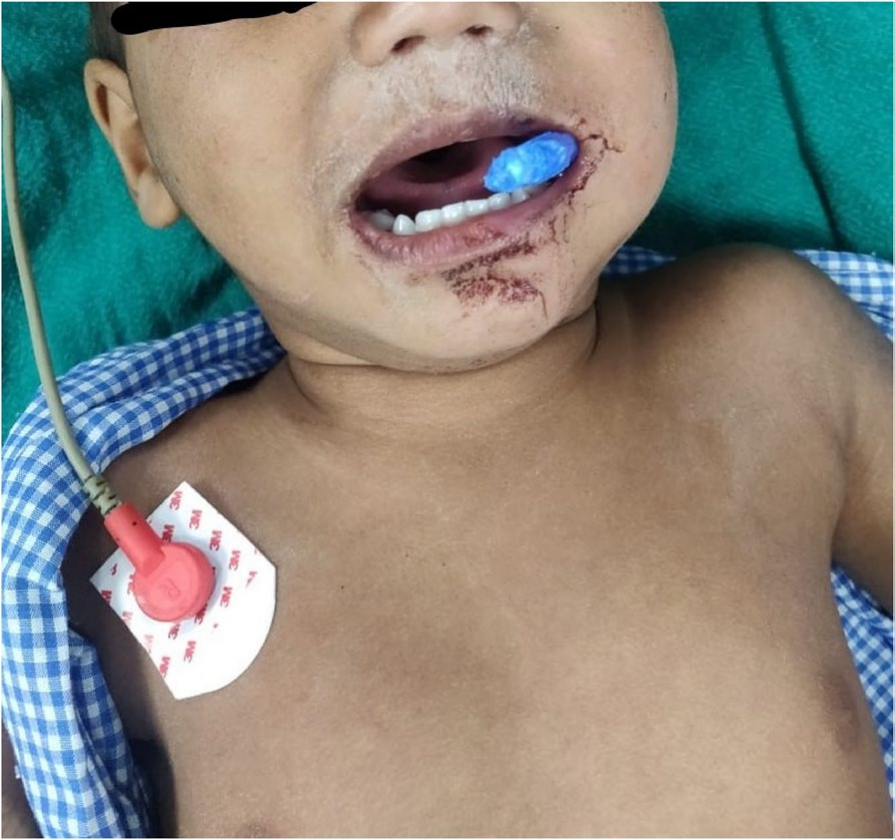
Distal end of toothbrush cut with bone cutter

Patient was NPO for last 9 h. 22 G IV (intravenous) cannula was already in place. Blood sugar was 47 mg/dl. IV dextrose was given to correct hypoglycemia. Difficult airway cart and surgeons were ready with the tracheostomy set to perform tracheostomy should the need arise. Patient was pre-medicated with 0.1 mg glycopyrrolate and wheeled into the OT and standard ASA monitors attached. Plan to induce GA using bag and mask ventilation followed by oral endotracheal intubation was made with tracheostomy as a backup. The part of the toothbrush protruding out was cut near the lips using a bone cutter by the orthopedics team (as ours is a multi-specialty emergency OT in the same complex) (Fig. 3). IV midazolam 1 mg, IV Fentanyl 20 μg were given to the patient followed by inhalational induction using a mixture of 50% nitrous oxide with 50% oxygen and incremental sevoflurane. Check ventilation was done and upon ensuring the ability to ventilate Inj Atracurium 5 mg IV was given and the patient was orally intubated using 4.5 mm I.D cuffed ETT. Oropharyngeal packing was done. IV Dexamethasone 2 mg was given. Maintenance of anesthesia was done using 50% nitrous oxide with 50% oxygen and 1% sevoflurane. The surgical procedure involved dilatation of entry wound and removal of the impacted portion of the toothbrush. At the end of the procedure, neuromuscular blockade was reversed uneventfully and the patient was extubated on table. Patient was shifted to pediatric ICU for observation of any signs of airway obstruction due to edema and then sent to the ward following which he was discharged on the 4th postoperative day.

## Discussion

Toothbrush although designed to be soft can rarely cause injury more commonly while performing some task while brushing or falling while brushing. The anesthetic concerns in such cases include inadequate history given by parents or child, non-cooperation by the child for airway examination and awake intubation, surgical bleeding in the oral cavity leading to a risk of aspiration under general anesthesia, and sharing of the airway between surgeon and the anesthesiologist.

In this case, one end of the toothbrush was lodged in the gingivobuccal sulcus while the other large part of it was jetting out of the mouth making it difficult to mask ventilate and intubate the patient. The major concerns in such an airway include difficulty in bag and mask ventilation due to the protruding foreign body, risk of aspiration due to the trickling of blood from the surgical site with concerns regarding pediatric airway. The patient can have hypoglycemia (seen in this patient also) and dehydration due to the inability to eat and drink. There is a possibility to land up in cannot ventilate cannot intubate situation in such a case.

Though our case involved impaction of toothbrush in the gingivobuccal sulcus, injuries of oropharynx involving soft palate, hard palate, tonsil, posterior pharyngeal wall and hard palate (Kupietzky 2000; Younessi and Alcaino 2007; Kosaki et al. 1992) have been reported, the most common site of lodgment of a foreign body being the left supra-tonsillar area. This has been attributed to right-handed dominance among patients (Kupietzky 2000). Penetrating injuries can cause various complications like injury to the carotid artery causing bleeding and thrombosis. This can cause cerebral symptoms after a period of 2–24 h, which is described as lucid interval. Therefore, the patients are observed for a period of 24–48 h to look for neurological deterioration such as vomiting, irritability, drowsiness, Horner’s syndrome, hemiparesis and upper airway obstruction which may develop in the hours following the injury. In addition, various complications like dehydration, dyspnea, mediastinitis, subcutaneous emphysema can occur with penetrating trauma of the oropharynx (Kosaki et al. 1992; Incollingo and Shevchenko 2007). Thus, level of consciousness, hydration, and vitals must be noted in pre-anesthetic examination. A CT scan head should be done to rule out damage or proximity to vascular and other vital structures. As the child had no compression symptoms so a CT scan was not needed here.

If the foreign body is close to vascular structures, movement of the foreign body must be avoided while securing the airway. Radiographic evidence of free air in retropharyngeal space or mediastinum warrants admission and close observation, broad-spectrum antibiotics and drainage if necessary (Incollingo and Shevchenko 2007).

Edem et al. described the anesthetic management of penetrating injury of the hard palate in the midline with a screw-driver. They described a three-man intubation technique where a larger size mask was used to ventilate the patient after inhalational induction using incremental doses of halothane and then one person applied cricoid pressure, another one did laryngoscopy using Macintosh blade and otorhinolaryngologist lifted the tongue away from laryngoscopic view using Magill forceps (Edem et al. 2017). Incollingo et al. described the use of a surgical rib cutter to cut a pen at the level of lips and then rapid sequence induction and intubation was done using Miller blade (Incollingo and Shevchenko 2007). Vemuru et al. described nasotracheal intubation and Park et al. described the use of a videolaryngoscope to secure the airway in such cases (Vemuru et al. 2014; Park et al. 2017). Fibreoptic-assisted intubation has also been described in the literature for securing the airway in such cases (Kumar et al. 2008; Gupta et al. 2010); however, awake fibreoptic is difficult in crying, irritable child and doing it under GA poses a risk of aspiration and loss of airway. Another good option is a videolaryngoscope, the size for 2-year old was not available in our emergency operation theatre.

Therefore, tracheostomy was kept as a backup while toothbrush was cut close to lips enabling the mask ventilation thus avoiding invasive airway management.

## Conclusions

Penetrating toothbrush injury is often seen in preschool children, which can present with dangerous complications, thus the approach to airway management needs to be individualized based on the site of impaction. Innovative approaches like the use of a cutter in this case helped prevent invasive airway in this case.

## Data Availability

Data sharing is not applicable to this article as no datasets were generated or analyzed during the current study.

## Abbreviations

ENT: Ear, nose and throat
ICU: Intensive care unit
ETT: Endotracheal tube
COVID-19: Coronavirus disease 2019
CT: Computed tomography
NPO: Nil per oral
